# Supplementary material for: The Replacement of Ground Corn with Sugar Beet in the Diet of Pasture-Fed Lactating Dairy Cows and Its Effect on Productive Performance and Rumen Metabolism
Source: Animals (Basel). 2022 Jul 28;12(15):1927. doi: 10.3390/ani12151927 (PMC9367446; doi:10.3390/ani12151927)
Supplement: Supplementary file 1 [file animals-12-01927-s001.zip › animals-1794803-supplementary.pdf]

Payment scheme of PROLESUR S.A. for raw milk of dairy farmers from Los Ríos region and districts of San Juan de la Costa, San Pablo and Puyehue from Los Lagos region

Prices and conditions of this payment scheme start on Abril 1st 2020.

1 **BASE PRICE**:
Base Price for a liter of milk with 3,0% w/v of fat and 3,0% w/v of crude protein will be CLP$124,58.

2 **FAT:** Differences with the 30 g considered in base price will be paid at CHLP$ 1,050/kg.

3 **PROTEIN**: Differences with the 30 g considered in base price will be paid at CHLP$ 5,550/KG.

4 **MILK SOLID BONUS:** The difference in the summation of milk fat and crude protein above 76.3 g/l, will be paid at a reason of CLP$ 1,150/kg

5 **BONUSES:** there are bonuses whose payment is calculated as a % of base price.

5.1 **Storage and refrigeration capacity:** the Company will buy only milk at a temperature below 6°C.

This bonus is calculated as follows: daily milk production (l) / tank capacity.

Index	Bonus
Below 1,0……	5,0%
Below 1,5……	0,0%
Above 1,5……	- 5,0%

5.2 Somatic cell count: Three analyses every 15 days.

Count (cel. / ml). Mean of 4 last fortnightly analyses.

Below 300.000.......	10 %
Below 400.000.......	8 %
Below 500.000.......	0 %
Below 800.000.......	-8 %
Above 800.000.......	-10 %

5.3 Bacterial count (bc): Two analyses every 15 days.

Count (bc / ml)

Below 30.000.......	14 %
Below 50.000.......	12 %
Below 80.000.......	10 %
Below 100.000.....	8 %
Below 300.000.....	0 %
Above 300.000......	-10 %

5.4 Farm free from bovine Brucellosis:
Farm declared free from brucelosis by the National Service of Agriculture and Livestock (SAG): 5%.

5.5 Farm free from bovine tubercolusis:
Farm declared free from tuberculosis by the National Service of Agriculture and Livestock (SAG): 5%.

5.6 Farm free from bovine enzootic leukosis:
Farm declared free from bovine enzootic leukosis by the National Service of Agriculture and Livestock (SAG): 4%.

5.7 Farm certified as PABCO A- (IT/3):
Farm certified by the by the National Service of Agriculture: 10%.

6 Growth bonus:

A bonus of CLP$10 / l will be paid to all farmers that increased the monthly milk production compared with the same month of previous year.

7 Winter bonus:

All milk delivered between 1st Abril and 31st August will receive additional CLP $27/l.

8 Bonus for stability of milk supply:

This bonus will be applied to the volumen of milk that at most double the volum produced in the two months of lowest milk production and will be paid as described in the following table:

	Relative Percentage to the months with lowest milk production		
Volume produced per year (million liters)	Below 20%	Greater than 20% and less than 70%	Above 70% and below 100%
Less than 4,5	CLP$/lt 23	CLP $/lt 18	CLP $/lt 13
Between 4,5 a 9,0	CLP $/lt 28	CLP $/lt 23	CLP $/lt 18
More than 9,0	CLP $/lt 33	CLP $/lt 28	CLP $/lt 23

9 Members of farmers societies will receive: CLP$ 1,20/l

10 Sustainality bonus: CLP$ 11/l

Due to the difficult times the country is facing, Prolesur will apply an extra bonus to support farmes from the compoany and ensure the normal delivery of milk.

Date: 1st April 2020.
